# A statistical approach to quantification of genetically modified organisms (GMO) using frequency distributions

**DOI:** 10.1186/s12859-014-0407-x

**Published:** 2014-12-14

**Authors:** Lars Gerdes, Ulrich Busch, Sven Pecoraro

**Affiliations:** Bavarian Health and Food Safety Authority (LGL), Veterinaerstr. 2, 85764 Oberschleissheim, Germany

**Keywords:** Genetically modified organism (GMO), Real-time PCR (qPCR), Quantification, Low-level presence (Regulation (EU) No 619/2011), Feed analysis, Simulation of frequency distribution

## Abstract

**Background:**

According to Regulation (EU) No 619/2011, trace amounts of non-authorised genetically modified organisms (GMO) in feed are tolerated within the EU if certain prerequisites are met. Tolerable traces must not exceed the so-called ‘minimum required performance limit’ (MRPL), which was defined according to the mentioned regulation to correspond to 0.1% mass fraction per ingredient. Therefore, not yet authorised GMO (and some GMO whose approvals have expired) have to be quantified at very low level following the qualitative detection in genomic DNA extracted from feed samples. As the results of quantitative analysis can imply severe legal and financial consequences for producers or distributors of feed, the quantification results need to be utterly reliable.

**Results:**

We developed a statistical approach to investigate the experimental measurement variability within one 96-well PCR plate. This approach visualises the frequency distribution as zygosity-corrected relative content of genetically modified material resulting from different combinations of transgene and reference gene Cq values. One application of it is the simulation of the consequences of varying parameters on measurement results. Parameters could be for example replicate numbers or baseline and threshold settings, measurement results could be for example median (class) and relative standard deviation (RSD). All calculations can be done using the built-in functions of Excel without any need for programming. The developed Excel spreadsheets are available (see section ‘Availability of supporting data’ for details). In most cases, the combination of four PCR replicates for each of the two DNA isolations already resulted in a relative standard deviation of 15% or less.

**Conclusions:**

The aims of the study are scientifically based suggestions for minimisation of uncertainty of measurement especially in —but not limited to— the field of GMO quantification at low concentration levels. Four PCR replicates for each of the two DNA isolations seem to be a reasonable minimum number to narrow down the possible spread of results.

**Electronic supplementary material:**

The online version of this article (doi:10.1186/s12859-014-0407-x) contains supplementary material, which is available to authorized users.

## Background

An important task of the official food and feed control in the European Union (EU) is to monitor the compliance of products with regulations related to labelling by laboratory analysis. In this context, food and feed samples are routinely screened for the presence of genetically modified organisms (GMO) or processed material derived thereof related to Regulation (EC) No 1829/2003 [1]. According to Regulation (EU) No 619/2011 [2], trace amounts of non-authorised GMO in feed are tolerated within the EU if certain prerequisites are met. Amongst other things, a pending application for authorisation with the European Food Safety Authority (EFSA [3]), a specific detection method validated by the EU Reference Laboratory for GM Food & Feed (EURL-GMFF [4]), and the availability of certified reference material are required. Tolerable traces must also not exceed the so-called ‘minimum required performance limit’ (MRPL), which was defined to correspond to 0.1% mass fraction of genetically modified material per ingredient. Currently, 26 GMO single and stacked events fulfil these requirements (EU Register of authorised GMO [5]; 27.10.2014). Because of this relatively new regulation, trace amounts of not yet authorised GMO (and some GMO whose approvals have expired) have to be quantified following the qualitative identification in genomic DNA extracted from feed samples. As the results of quantitative analysis can imply severe legal and financial consequences for producers or distributors of feed, the quantification results need to be reliable.

Although new techniques —like for example digital PCR [6]— have appeared in the field of GMO analysis, the most common technique used today for routine analysis on the presence of GMO is (quantitative) real-time PCR (qPCR) with hydrolysis probes. It is still the method of choice because of its high specificity, and its closed amplification system in sealed microtitre plates that minimises carryover risks; it also offers the possibility for subsequent quantification of GMO contents with the same experimental principle. In fact, real-time thermal cyclers are already available in most laboratories. Additionally, all official quantitative detection methods published by the EURL-GMFF are so far based on this chemistry [4].

Generally, random and systematic errors influence all measurement results [7]. This holds also true for quantification of GMO at all levels —not only at 0.1% [8]. Quantification of GMO adds further obstacles to the measuring procedure: After quantitative real-time PCR of both the transgene and a species-specific reference gene, the corresponding mass fraction has to be calculated considering the (assumed) zygosity of the plant tissue(s) and plant species under investigation [9].

To shine new light on the challenging quantification tasks (at the limit of quantification) following the introduction of Regulation (EU) No 619/2011 [2], we developed a statistical approach to investigate the experimental measurement variability within one 96-well qPCR plate. This approach visualises the frequency distribution as relative content of genetically modified material resulting from different combinations of transgene and reference gene Cq values. One application is the simulation of the consequences of varying parameters on measurement results. Parameters could be for example replicate numbers or baseline and threshold settings, measurement results could be for example median (class) and relative standard deviation (RSD). All calculations can be done using the built-in functions of Excel without any need for programming. We envision scientifically based suggestions for minimisation of uncertainty of measurement especially in —but not limited to— the field of GMO quantification at low concentration levels.

## Methods

### Samples

Certified reference materials (ground plant material) for GMO events soy 305423, 356043 and maize NK603, MON 863, 59122 were purchased from IRMM (Geel, Belgium), material for event soy MON 89788 was purchased from AOCS (Urbana, USA). Dual- or multi-target target plasmids for calibration of events soy 305423 and maize MON 810 were designed in-house and subsequently synthesised, propagated, purified, and linearized by Eurofins-MWG (Ebersberg, Germany). Low GMO percentage DNA solution for soy MON 89788 was mixed from isolated DNA of 100% material according to the method in Annex 3 of the guidance document [10].

### DNA extraction

Genomic DNA (gDNA) was extracted from ground material with the Maxwell 16 instrument (Promega, Mannheim, Germany) using a modified protocol [11]. Some batches of the isolated gDNA were further purified with DNA Extractor Cleaning Columns Kit (Eurofins-GeneScan). Quantity of gDNA was estimated using the Quant-iT PicoGreen dsDNA reagent (LifeTechnologies, Darmstadt, Germany), or the NanoDrop 1000 instrument (Thermo Scientific, Wilmington, USA).

### Oligonucleotides

Oligonucleotide primers and hydrolysis probes were synthesised by TIB Molbiol (Berlin, Germany), Eurofins-MWG, or Life Technologies (formerly Applied Biosystems, Carlsbad, USA). Oligonucleotide sequences for the events quantified in this study were obtained from the official EU method collection [4].

### Real-time PCR procedure

Real-time PCR was performed with either a 7900HT or ViiA7 real-time PCR thermal cycler (both Life Technologies). For hydrolysis probes, ABI Universal mastermix with UNG (Life Technologies) was used. The total reaction volume was 25 μL, containing 1x mastermix, primers and probes as stated above in section Oligonucleotides [4]. After manual setup, 20 μL of this mixture and subsequently 5 μL sample DNA were distributed automatically into the wells of 96-well reaction plates (MicroAmp, Life Technologies) using a pipetting robot (epMotion 5070, Eppendorf, Hamburg, Germany, or Piro, Dornier-LTF, Lindau, Germany) at constant cooling to 4°C. After sealing with adhesive optical film (MicroAmp, Life Technologies), the following temperature profile was used for real-time PCR: 120 s 50°C, 600 s 95°C, and 50 cycles of 15 s 95°C, and 60 s 60°C. Ramping on both machines was adapted to +0.8°C/s and –1.6°C/s (corresponding to ‘9600 emulation’ on the 7900HT).

### Data analysis

Data were analysed with the corresponding software of the real-time cyclers: SDS 2.4 for the 7900HT and ViiA7 Software v1.1 for the ViiA7, respectively. Fluorescence data were normalised to the ROX signal (Rn), and a baseline signal (either automatic or cycles 3–15) was subtracted (dRn). Threshold was set automatically or manually either to 0.05 or 0.1. Based on previous experiences, five setups for baseline/threshold were routinely chosen (Table 1). Separate standard curves for transgene and reference gene were used to estimate copy numbers from Cq values obtained for the samples. Copy numbers were transformed to GMO percentages according to the published approximation given in the ENGL (European Network of GMO Laboratories) guidance document [10]. Datasets for each of the five baseline/threshold setups (Table 1) were exported from the cycler software to Excel (Microsoft, Unterschleissheim, Germany). A special Excel spreadsheet was developed that automatically collects data from the chosen sheet (and thus baseline/threshold setup). Switching between the different setups can be done by mouse click via spin buttons. (The spreadsheet is available; see section ‘Availability of supporting data’ for details).

**Table 1:** **Baseline/threshold settings**

	**Setting 1**	**Setting 2**	**Setting 3**	**Setting 4**	**Setting 5**
Baseline	automatic	automatic	automatic	3–15	3–15
Threshold	automatic	0.1	0.05	0.1	0.05

The table shows the five setups for baseline/threshold settings that were employed in this study. Baseline was either set automatically by the corresponding software of the real-time cycler (‘automatic’), or set to cycles 3 to 15 (‘3–15’). The threshold was either set automatically by the corresponding software of the real-time cycler (‘automatic’), or set to one of two different normalised dRn values (‘0.1’ or ‘0.05’).

### Frequency distribution

**Figure 1:**
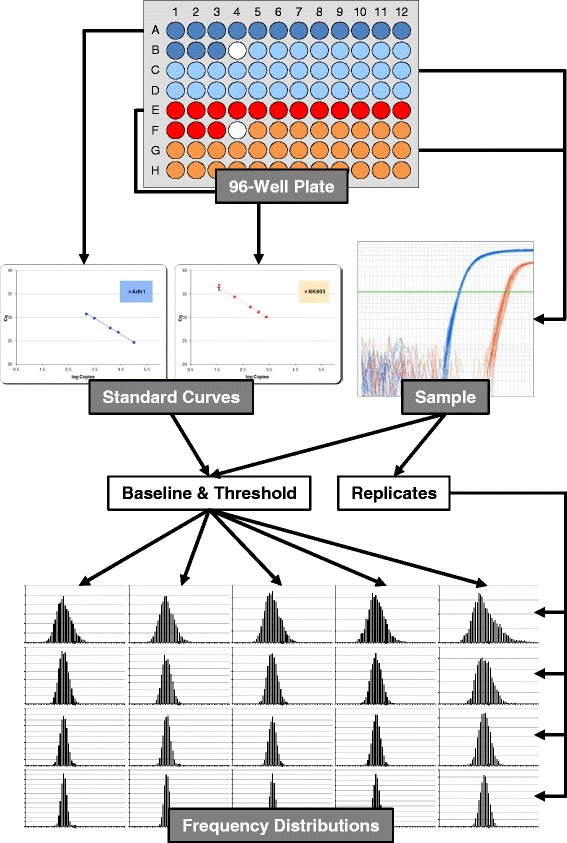
**Flowchart for generation of frequency distributions.** Illustrated flowchart for the generation of several frequency distributions starting from a single experimental 96-well plate. Transgene (red) and reference gene (blue) are analysed via real-time PCR in separate wells. The light coloured wells contain reactions for 32 replicates of the sample DNA to be measured. Transgene and reference gene copy numbers are extrapolated from corresponding standard curves (dark colours). The white well depicts negative control. For each number of possible transgene or reference gene replicates (considered in two isolations each, i.e. 2× 2, 2× 3, 2× 4, 2× 5,…, 2× 16), copy numbers are arranged in 5000 combinations by chance. The resulting 5000 GMO percentages are divided into frequency classes and depicted. For each number of replicates, a corresponding frequency distribution with its statistical parameters can be visualised. Effects of different baseline/threshold settings are shown from left to right, increasing replicate numbers from top to bottom, respectively.

**Figure 2:**
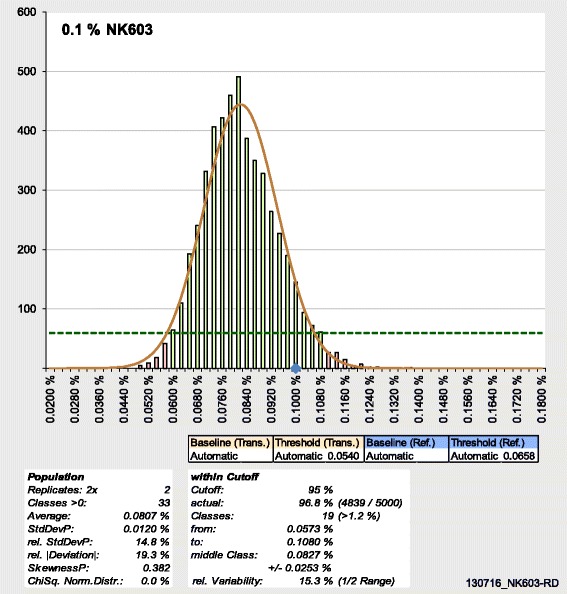
**Frequency distribution for quantification of maize NK603.** Exemplary frequency distribution taken from the dynamic Excel spreadsheet developed in this study. Isolated genomic DNA from certified reference material for maize event NK603 (0.1% [w/w]) was quantified with the novel statistical approach (Figure 1). Figure 2 shows the frequency distribution for automatic setting of baseline and threshold and for 2× 2 replicates (i.e. four randomly chosen copy numbers for transgene were analysed with four randomly chosen copy numbers for reference gene). Columns represent the number of values in the corresponding class (represented by the upper limit given on the horizontal axis). The blue diamond marks the nominal GMO content of the reference material. The corresponding Gaussian normal distribution is shown as brown line. All classes that reach the cut-off (dashed horizontal line) contribute together to at least 95% of all values (green columns; here 96.8%, i.e. 4839/5000). Statistical parameters are shown below the graph: on the left for the values of the entire population ([rel.] StdDevP: [relative] standard deviation of the population; relative |Deviation|: absolute difference average minus nominal value divided by nominal value; SkewnessP: skewness of the population; ChiSq. Norm.Distr.: simplified χ^2^ test for Gaussian normal distribution with 5% level of significance), on the right for the classes within the cut-off (middle class: upper limit of last included class plus upper limit of first included class divided by 2; ±: range from upper limit of last included class to upper limit of first included class divided by 2).

On a single 96-well plate 2× 32 replicates of the same sample where analysed for transgene and reference gene, respectively. For plate layout refer to Figure 1. Data from one experimental run was analysed with five different baseline/threshold settings (Table 1) and copied in five separate sheets in the same Excel template. Using the built-in Excel functionality a data table (formerly known as multi operation) was filled with 5000 random combinations of 32 wells for reference gene and 32 wells for transgene. (Note: The random combinations were created once and then used for all analysis in order to be able to re-create the exact same calculations and thus document the results. Calculation of the whole data table for 2x 2 to 2x 16 replicates and a single baseline/threshold setting takes around 30 minutes with our hardware and Excel 2010). The corresponding 5000 GMO percentage values for each replicate number were copied into a second Excel spreadsheet. (The spreadsheet is available; see section ‘Availability of supporting data’ for details). The results were distributed to frequency categories and visualised using built-in diagrams (compare for example Figure 2). Switching between the different settings (baseline/threshold) and parameters (number of classes, replicates, cut-off, GMO) can be done by mouse click via spin buttons. All data analysis and visualisations were implemented without programming (VBA), using entirely the standard Excel functions with the rS1.Method [12] as explained for Excel 2010 [13].

### Statistics

The calculations presented here assume that the 5000 values for GMO percentage generated from the 32 technical replicates for reference gene and transgene, respectively, represent the population and not a mere sample from the population. This is why the corresponding functions for populations were used (e.g. STDEV**P**). Average and standard deviation of the population were calculated using the built-in Excel functions (AVERAGE, STDEVP). The empirical skewness of the population was calculated as $\upsilon =\frac{1}{n}{{\displaystyle {\sum}_{i=1}^n\left(\frac{x_i-\overline{x}}{s}\right)}}^3$ with n as population size, x as GMO percentage values, and s as standard deviation of the population of GMO percentage values. The simplified χ^2^ test for normal distribution was done as follows: χ^2^ was calculated for comparison of the observed distribution with the Gaussian normal distribution (${x}^2={\displaystyle \sum \frac{d^2}{m}}\;\mathrm{with}\kern0.35em d=n-m$ with observed values n and expected values m for the respective classes [14]) in a simplified way without combining the marginal classes when < 10. The Excel function CHIDIST was used to estimate the probability that the observed distribution is a Gaussian normal distribution; calculated as CHIDIST(χ^2^,degrees of freedom). The result is displayed in percentage.

## Results

### Experimental setup, calculation and data analysis

We developed a statistical method to investigate the experimental measurement variability associated with the quantification of GMO content by real-time PCR. The developed semi-automatic report sheet visualises the frequency distribution of GMO percentages resulting from different combinations of transgene and reference gene Cq values. The approach focuses on the variation within a single 96-well PCR plate (Figure 1). To our knowledge, the effects of this intra-plate variability have not been studied yet.

GMO quantification was done using the standard curve method, comparing Cq values from sample wells with regression lines obtained from Cq values from known copies of reference gene or transgene, respectively. As standard curves consisted of five points in triplicates (2× 15 wells), after deduction of a negative control (2× 1 well), samples could be analysed in 32 replicates (2× 48 – 2× 15 – 2× 1 = 2× 32; compare plate set-up in Figure 1). DNA from food or feed samples is routinely isolated in duplicates in our laboratory, resulting in two isolations for each sample. The 32 wells could thus be filled with a maximum of two isolations with 16 replicates each. The number of replicates actually used for calculation of GMO content can be varied from four (2× two wells) to 32 (2× 16), resulting in 15 replicate settings (2× 2, 2× 3,…, 2× 16). As baseline/threshold settings for the interpretation of real-time PCR amplification curves determine the Cq value, different (routinely five) settings for baseline/threshold were employed (Table 1). All Cq values were exported to Excel. Data analysis and visualisation were implemented without programming (VBA), using entirely the standard Excel functions with the rS1.Method [12]. Extrapolated copy numbers were arranged in 5000 combinations by chance for each number of possible transgene or reference gene replicates. The resulting 5000 GMO percentages were divided into frequency classes. For each number of replicates and baseline/threshold, a corresponding frequency distribution with its statistical parameters can be visualised (Figure 2). Each column in the graph represents the number of values in the corresponding class. Optionally, the corresponding Gaussian normal distribution can be displayed for visual comparison (the numerical result of a simplified χ^2^ test is given below, together with the skewness of the distribution). The statistical parameters are calculated either for the entire population of results (GMO concentrations), or for the classes within a selectable cut-off (e.g. 95% of the population). The relative deviation is employed as a measure for trueness. Further examples for frequency distributions from maize and soy quantifications are given in the Supplementary data (Additional file 1: Figure S1, Additional file 2: Figure S2, Additional file 3: Figure S3, Additional file 4: Figure S4 and Additional file 5: Figure S5).

### Different settings, number of replicates

**Figure 3:**
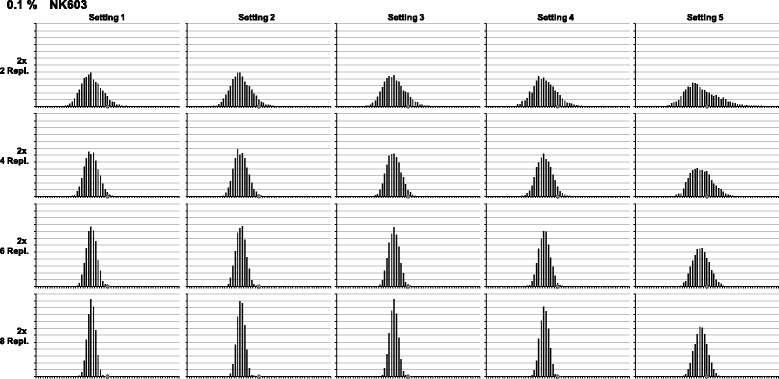
**Frequency distributions for quantification of maize NK603.** Comparative overview over 20 exemplary frequency distributions resulting from a single experimental 96-well plate for the quantification of maize event NK603 (compare Figure 2). Effects of different baseline/threshold settings (Table 1) are shown from left to right, increasing replicate numbers from top to bottom, respectively. The grey diamond marks the nominal GMO content of the reference material.

**Figure 4:**
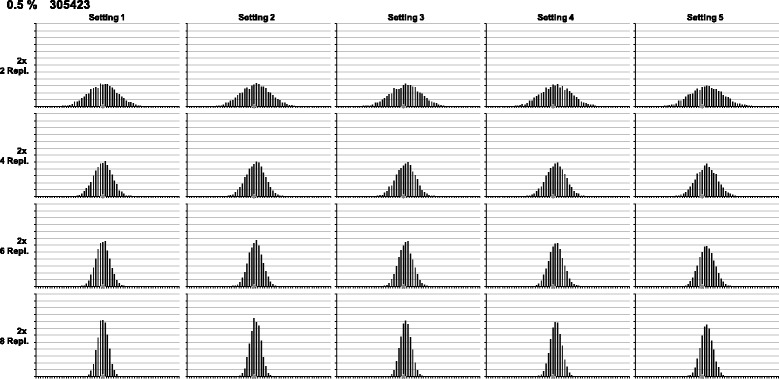
**Frequency distributions for quantification of soy 305423.** Comparative overview over 20 exemplary frequency distributions resulting from a single experimental 96-well plate for the quantification of soy event 305423 (compare Additional file 3: Figure S3). Effects of different baseline/threshold settings (Table 1) are shown from left to right, increasing replicate numbers from top to bottom, respectively. The grey diamond marks the nominal GMO content of the reference material.

Frequency distributions are visualised using dynamic Excel spreadsheets: parameters (e.g. baseline/threshold, number of replicates) can be adjusted using spin buttons resulting in changes being directly visible in statistical values and frequency diagrams (Figure 2). To show at least some of these dynamics on the printed page, multiple graphs were generated, each one showing the multitude of possible frequency distributions (Figures 3 and 4). The comparative overview over 20 exemplary frequency distributions for the quantification of maize event NK603 (Figure 3) is based completely on experimental data from a single 96-well plate (compare Figure 2). The same holds true for the comparative overview based on the quantification of soy event 305423 (compare Additional file 3: Figure S3). The underlying effects of different baseline/threshold settings are presented from left to right, and the impact of increasing replicate numbers from top to bottom, respectively. As expected, with increasing replicate numbers, the distribution of the population is narrowing down (Figures 3 and 4). Finally, when all replicates are taken together, variation is levelled, and all values fall into one or two classes (data not shown). Further overviews for frequency distributions from maize and soy quantifications are given in the Supplementary data (Additional file 6: Figure S6, Additional file 7: Figure S7, Additional file 8: Figure S8 and Additional file 9: Figure S9).

By visual inspection of these overviews, an approximation of the population shape to the bell-shaped Gaussian normal distribution is obvious. Even quite skewed distributions, e.g. in Figure 3 Setting 5, become regularly bell-shaped when more replicates were taken into account. Mathematically, empirical skewness in this example drops from 0.917 (2× 2 replicates) to 0.038 (2× 8 replicates). For comparison, skewness for Setting 1 in the same measurement drops from 0.382 to 0.006 (Table 2). Further examples for reductions in skewness can be found in the supplementary data (Additional file 10: Tables S1-S5).

**Table 2:** **Empirical skewness (0.1% NK603, Figure** 3 **)**

**Replicates**	**Setting 1**	**Setting 2**	**Setting 3**	**Setting 4**	**Setting 5**
2× 2	0.382	0.387	0.388	0.413	0.917
2× 4	0.232	0.242	0.234	0.192	0.388
2× 6	0.087	0.082	0.094	0.057	0.167
2× 8	0.006	−0.009	0.007	0.015	0.038

The table shows the empirical skewness (for calculation see section ‘Methods: Statistics’) yielded for each of the five settings (Table 1) and taking two DNA isolations (‘2×’) with four different exemplary numbers of PCR replicates into account (‘2’, ‘4’, ‘6’, or ‘8’).

Although by visual inspection the populations appear normally distributed, the mathematical probability was tested for by a simplified χ^2^ test (see Methods for details). In the same example as for the drop in skewness (Figure 3 Setting 5), the fit to a Gaussian normal distribution rises only from 0.0% (2× 2 replicates) to 2.3% (2× 8), whereas in Setting 1 the rise is from 0.0% to 84.5% (Table 3). Visual inspection is sometimes quite in contrast with the χ^2^ test results (compare 2× 8 replicates in Table 3). Further test results for normal distributions are listed in the supplementary data (Additional file 10: Tables S6-S10).

**Table 3:** **χ** ^**2**^ **test for normal distribution (0.1% NK603,** Figure 3 **)**

**Replicates**	**Setting 1**	**Setting 2**	**Setting 3**	**Setting 4**	**Setting 5**
2× 2	0.0%	0.0%	0.0%	0.0%	0.0%
2× 4	0.0%	0.0%	0.0%	0.0%	0.0%
2× 6	5.8%	7.6%	8.2%	12.4%	0.0%
2× 8	84.5%	8.1%	73.6%	4.2%	2.3%

The table shows the results for the simplified χ^2^ test (for calculation see ‘Methods: Statistics’) yielded for each of the five settings (Table 1) and taking two DNA isolations (‘2×’) with different exemplary numbers of PCR replicates into account (‘2’, ‘4’, ‘6’, or ‘8’).

**Figure 5:**
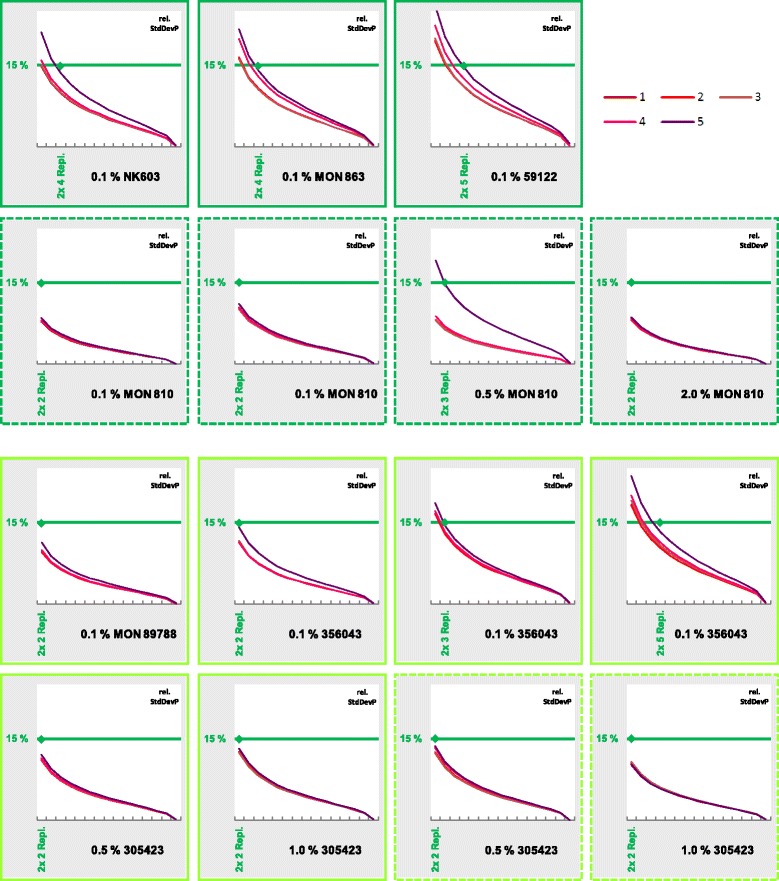
**Effect of number of replicates on relative standard deviation.** Each partial figure shows the effect of different numbers of combined replicates (abscissa) on the relative standard deviation of the population (ordinate). Values for five different settings of baseline/threshold (1–5; Table 1) are depicted. The green diamond marks the number of replicates with which a maximum of 15% relative standard deviation is achieved (for all settings). The titles state the name of the event and the nominal value of the measured reference material. Each partial figure is based on experimental data from a single 96-well plate. Dashed frames indicate experiments with plasmidic standard curves (plasmids were linearized before use).

The number of combined replicates has an effect on the spread of the values of the population. Relative standard deviation of the population (rel. StdDevP) was used as a criterion for this spread. In most measurements, the combination of 2× 4 replicates results in a rel. StdDevP of 15% or less (Figure 5). In the cases where 2× 5 replicates where needed to reach 15%, this was caused by one setting only (0.1% 59122, 0.1% 356043 in Figure 5). 15% was chosen as an arbitrary threshold. It is well below the 25% relative repeatability standard deviation required by regulation in the EU [2]. If the spread of possible values from a single plate exceeds 15%, it might be difficult to keep below 25% under repeatability conditions.

**Figure 6:**
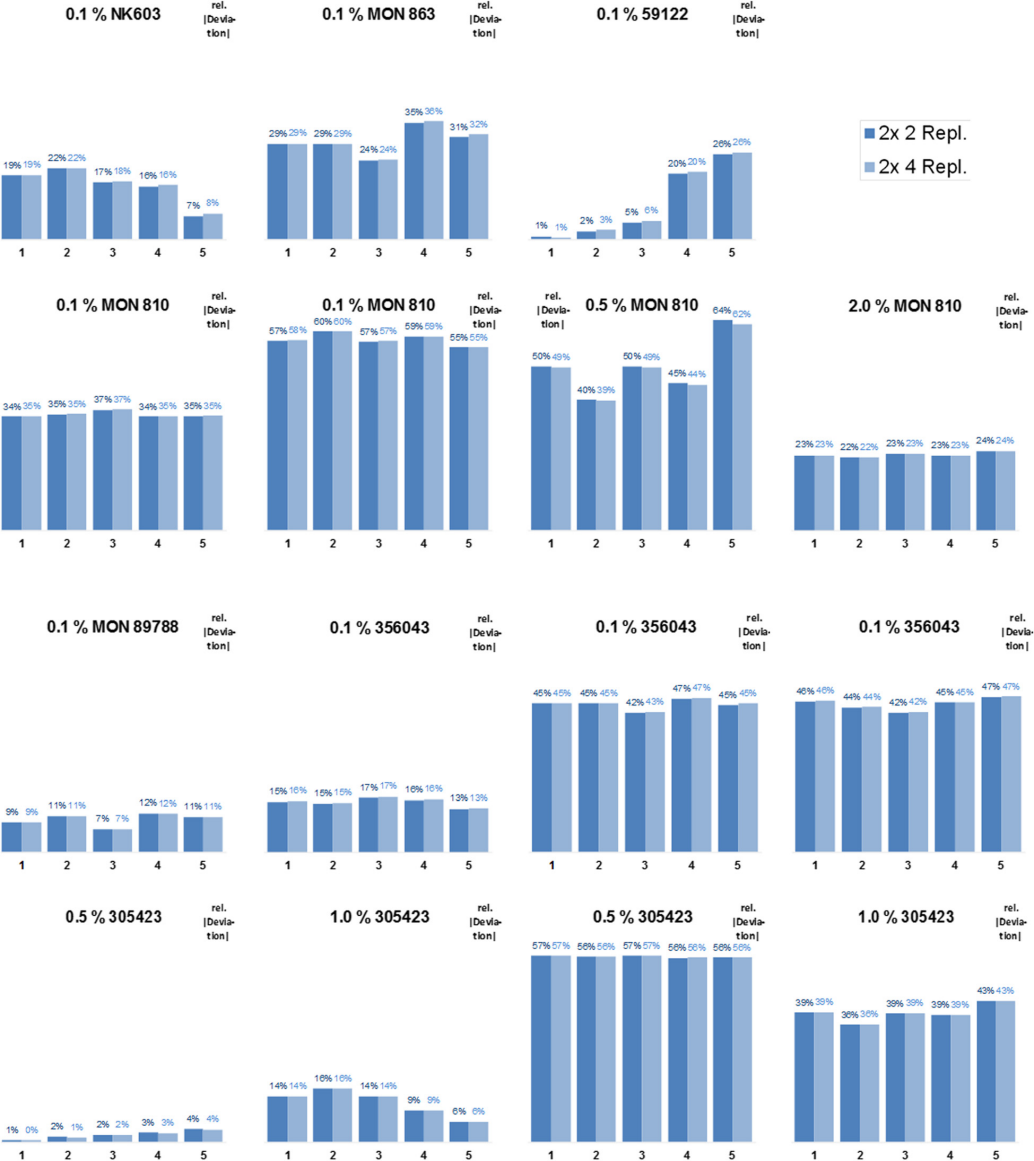
**Effect of baseline/threshold setting on relative deviation from nominal value.** Each partial figure depicts the relative absolute deviation from the nominal value of the measured reference material (|arithmetic mean – nominal value|/nominal value) for five different settings of baseline/threshold (1–5; Table 1) and two numbers of replicates (2x 2 and 2x 4). The headings state the name of the event and the nominal value of the measured reference material. Each partial figure is based on experimental data from a single 96-well plate.

The spread of the values is only one aspect, another point is even more important. If the results cluster closely around a mean it is still interesting to know how well this mean represents the true value. The relative absolute deviation from the nominal value (|arithmetic mean – nominal value|/nominal value) was used to quantify the trueness of the obtained values (Figure 6). Though the spread of the values of the population narrows down with increasing replicates (Figure 5), the deviation of the mean from the associated nominal true value does not (Figure 6). Interestingly, in some cases the baseline/threshold settings have a distinct influence on the trueness of the result (0.1% 59122 in Figure 6 and Additional file 6: Figure S6).

## Discussion

The introduction of Regulation (EU) No 619/2011 [2] poses a challenging analytical task on GMO testing laboratories in the EU, namely to accurately quantify around 0.1% GM material per ingredient, which is close to the limit of quantification (LOQ). Therefore, we developed a statistical approach to investigate the experimental measurement variability. The variability within one single 96-well PCR plate was analysed for several GMO percentages including —but not limited to— 0.1%. Frequency distributions of the results could be analysed visually, and data for corresponding statistical parameters of the population like skewness and fit to Gaussian normal distribution were collected.

To our knowledge, no similar analysis of intra-plate variation and effects of baseline and threshold settings has been published for GMO analysis so far. Especially the setting of baseline and threshold can be crucial to the results. Some researchers set either baseline or threshold settings or both automatically (e.g. [15-19]). To the authors opinion it is advisable in this cases to re-check the results obtained also with manual settings. As the underlying built-in algorithms of the instrument software might change with a new software version this should also be considered. Other researchers may more rely on experience when setting baseline and threshold; these manual settings are sometimes reported in the Methods section (e.g. [20]), sometimes not. Nevertheless, a threshold of for example 0.1 might work fine with one specific assay. The authors recommend that a generally applied threshold should be reconsidered carefully especially when running an assay on another instrument. Often results in scientific publications on GMO quantification are given without numerical information about the software settings (e.g. [21-24]). Sometimes it is mentioned that baseline and threshold were set according to the manufacturer’s or other published instructions (e.g. [25]). Our statistical approach on the one hand draws attention to the baseline and threshold settings and on the other hand gives experimentally based clues related to the practical importance of these settings. Researchers applying the developed spreadsheet can reproduce selected experiments in their laboratories in order to get a good impression about the effects of settings on their assays and instrumentation. This could include the shape of the generated distributions, either visual, or, by descriptive mathematical means of skewness and fit to Gaussian normal distribution. According to the authors opinion and experience absolute skewness values above 0.5 can hint to outlying Cq values that distort the expected Gaussian normal distribution. To some extent skewness can be reduced by careful selection of baseline/threshold settings.

The methods for GMO detection and quantification validated by the EURL-GMFF should show an RSD_r_ value of maximum 25% [9,26]. When verifying a new method in the lab this may be difficult to achieve, at least for the level of 0.1% GM material. Our test scheme allows checking for the extent of the variation that is based on the spread of intra-plate Cq values associated with the assay. The consequences of variations in Cq values under different baseline and threshold settings can be easily assessed and correlated to different replicate numbers. Especially when quantifying low GMO percentages at around 0.1%, an increase of PCR replicates might be considered to decrease the possible spread of results. Our data (Figure 5) suggests that two DNA isolations with four PCR replicates (2× 4) seems to be a reasonable minimum number for this aim.

As in some cases the baseline and threshold settings have a distinct influence on the trueness of the result (0.1% 59122 in Figure 6 and Additional file 6: Figure S6) we recommend to test different settings during method verification. It also can be advisable to check the assay for possible effects when changing instrumentation (thermal cyclers) or suppliers of chemicals (mastermix, oligonucleotide primers or probes).

The developed Excel spreadsheets support (raw) data from three different qPCR systems: Agilent Mx3005P, LifeTechnologies 7900HT and ViiA7. When necessary, further systems could be integrated, given that the instrument software is capable of exporting into a suitable format (e.g. .csv, .txt, .xls, .xlsx). Co-operation with other labs to generate more datasets could be very beneficial. The number of 5000 random combinations is due to the limitations in available soft- and hardware: calculations with Excel 2003 took around three hours; with Excel 2010 it still takes around 30 minutes for calculation of one baseline/threshold setting, resulting in a total time of 2.5 hours per dataset with five settings. It might be interesting to expand the number of combinations with even more powerful computers.

We envision the presented statistical approach to be used by researchers to investigate the effects of intra-plate spread in assays typically performed in their laboratories. With these datasets from several laboratories, the statistical conclusions could be expanded on a reproducibility and repeatability basis.

## Conclusions

The developed statistical approach allows simulation of the experimental measurement variability, especially concerning baseline/threshold settings and the number of replicates combined for analysis. Researchers are encouraged to reproduce the experimental setup, use the developed Excel spreadsheets and draw conclusions about the corresponding performance of their own assays. Analysis of data from our laboratory suggests that two isolations with four PCR replicates each (eight in total) seems to be a reasonable minimum number to decrease the possible spread of results especially when quantifying GMO percentages around 0.1%.

### Availability of supporting data

Supplementary figures and tables are available via BMC Bioinformatics.

The three developed Excel files with the presented data were deposited at LabArchives (http://www.labarchives.com/) for download as zip files via the DOI (http://www.doi.org/): 130716_NK603-RD_Quant-Statisti k_v4-02-1.zip (DOI 10.6070/H44B2Z9H), 01_Statistik-Zusammenfassung_5 000_v4-06.zip (DOI 10.6070/H40K26JQ), 02_Statistik-Zusammenfassung_5 000_v4-06.zip (DOI 10.6070/H4VT1Q2F).

## Additional files

Additional file 1: Figure S1. Frequency distribution for quantification of maize 59122. Exemplary frequency distribution taken from the developed dynamic Excel spreadsheet. Isolated genomic DNA from certified reference material for maize event 59122 (0.1% [w/w]) was quantified with the novel statistical approach. For explanations, see Figure 2. Additional file 2: Figure S2. Frequency distribution for quantification of maize MON 863. Exemplary frequency distribution taken from the developed dynamic Excel spreadsheet. Isolated genomic DNA from certified reference material for maize event MON 863 (0.1% [w/w]) was quantified with the novel statistical approach. For explanations, see Figure 2. Additional file 3: Figure S3. Frequency distribution for quantification of soy 305423. Exemplary frequency distribution taken from the developed dynamic Excel spreadsheet. Isolated genomic DNA from certified reference material for soy event 305423 (0.5% [w/w]) was quantified with the novel statistical approach. For explanations, see Figure 2. Additional file 4: Figure S4. Frequency distribution for quantification of soy MON 89788. Exemplary frequency distribution taken from the developed dynamic Excel spreadsheet. Isolated genomic DNA from self-mixed reference material for soy event MON 89788 (0.1% [cp/cp]) was quantified with the novel statistical approach. For explanations, see Figure 2. Additional file 5: Figure S5. Frequency distribution for quantification of soy 356043. Exemplary frequency distribution taken from the developed dynamic Excel spreadsheet. Isolated genomic DNA from certified reference material for soy event 356043 (0.1% [w/w]) was quantified with the novel statistical approach. For explanations, see Figure 2. Additional file 6: Figure S6. Frequency distributions for quantification of maize 59122. Comparative overview over 20 exemplary frequency distributions resulting from a single experimental 96-well plate for the quantification of maize event 59122 (compare Additional file 6: Figure S6). Effects of different baseline/threshold settings (Table 1) are shown from left to right, increasing replicate numbers from top to bottom, respectively. The grey diamond marks the nominal GMO content of the reference material. Additional file 7: Figure S7. Frequency distributions for quantification of maize MON 863. Comparative overview over 20 exemplary frequency distributions resulting from a single experimental 96-well plate for the quantification of maize event MON 863 (compare Additional 7: Figure S7). Effects of different baseline/threshold settings (Table 1) are shown from left to right, increasing replicate numbers from top to bottom, respectively. The grey diamond marks the nominal GMO content of the reference material. Additional file 8: Figure S8. Frequency distributions for quantification of soy MON 89788. Comparative overview over 20 exemplary frequency distributions resulting from a single experimental 96-well plate for the quantification of soy event MON 89788 (compare Additional file 8: Figure S8). Effects of different baseline/threshold settings (Table 1) are shown from left to right, increasing replicate numbers from top to bottom, respectively. The grey diamond marks the nominal GMO content of the reference material. Additional file 9: Figure S9. Frequency distributions for quantification of soy 356043. Comparative overview over 20 exemplary frequency distributions resulting from a single experimental 96-well plate for the quantification of soy event 356043 (compare Additional file 9: Figure S9). Effects of different baseline/threshold settings (Table 1) are shown from left to right, increasing replicate numbers from top to bottom, respectively. The grey diamond marks the nominal GMO content of the reference material. Additional file 10: Table S1. Empirical skewness (0.5% 305423, Figure 4). **Table S2:** Empirical skewness (0.1% 59122, Additional file 6: Figure S6). **Table S3:** Empirical skewness (0.1% MON 863, Additional file 7: Figure S7). **Table S4:** Empirical skewness (0.1% MON 89788, Additional file 8: Figure S8). **Table S5:** Empirical skewness (0.1% 356043, Additional file 9: Figure S9). **Table S6:** $\chi$ ^2^ test for normal distribution (0.5% 305423, Figure 4). **Table S7:** $\chi$ ^2^ test for normal distribution (0.1% 59122, Additional file 6: Figure S6). **Table S8:** $\chi$ ^2^ test for normal distribution (0.1% MON 863, Additional file 7: Figure S7). **Table S9:** $\chi$ ^2^ test for normal distribution (0.1% MON 89788, Additional file 8: Figure S8).

## Abbreviations

ChiSq. Norm.Distr: χ^2^ test for Gaussian normal distribution
cp/cp: (gene) copy per (gene) copy
Cq: Quantification cycle (previously known as the threshold cycle (Ct), crossing point (Cp), or take-off point (TOP)
DNA: Deoxyribonucleic acid
EC: European Commission
EFSA: European Food Safety Authority
ENGL: European Network of GMO Laboratories
EU: European Union
EURL-GMFF: European Reference Laboratory for GM Food and Feed
gDNA: Genomic DNA
GM: Genetically modified
GMO: Genetically modified organism
L: Liter
LLP: Low-level presence
MRPL: Minimum required performance limit
MS: Microsoft
No: Number
qPCR: (quantitative) real-time PCR
PCR: Polymerase chain reaction
Ref: Reference gene
Rel: Relative
Repl: Replicates
RSD: Relative standard deviation
RSD_r_: RSD under repeatability conditions
RSD_R_: RSD under reproducibility conditions
SkewnessP: Empirical skewness of the population
StdDevP: Standard deviation of the population
Trans: Transgene
UNG: Uracil-DNA-glycosylase
VBA: Visual basic for applications
w/w: Weight per weight
