# Supplementary figures and images for: A statistical approach to quantification of genetically modified organisms (GMO) using frequency distributions
Source: BMC Bioinformatics. 2014 Dec 14;15(1):407. doi: 10.1186/s12859-014-0407-x (PMC4279603; doi:10.1186/s12859-014-0407-x)

## Slide 1
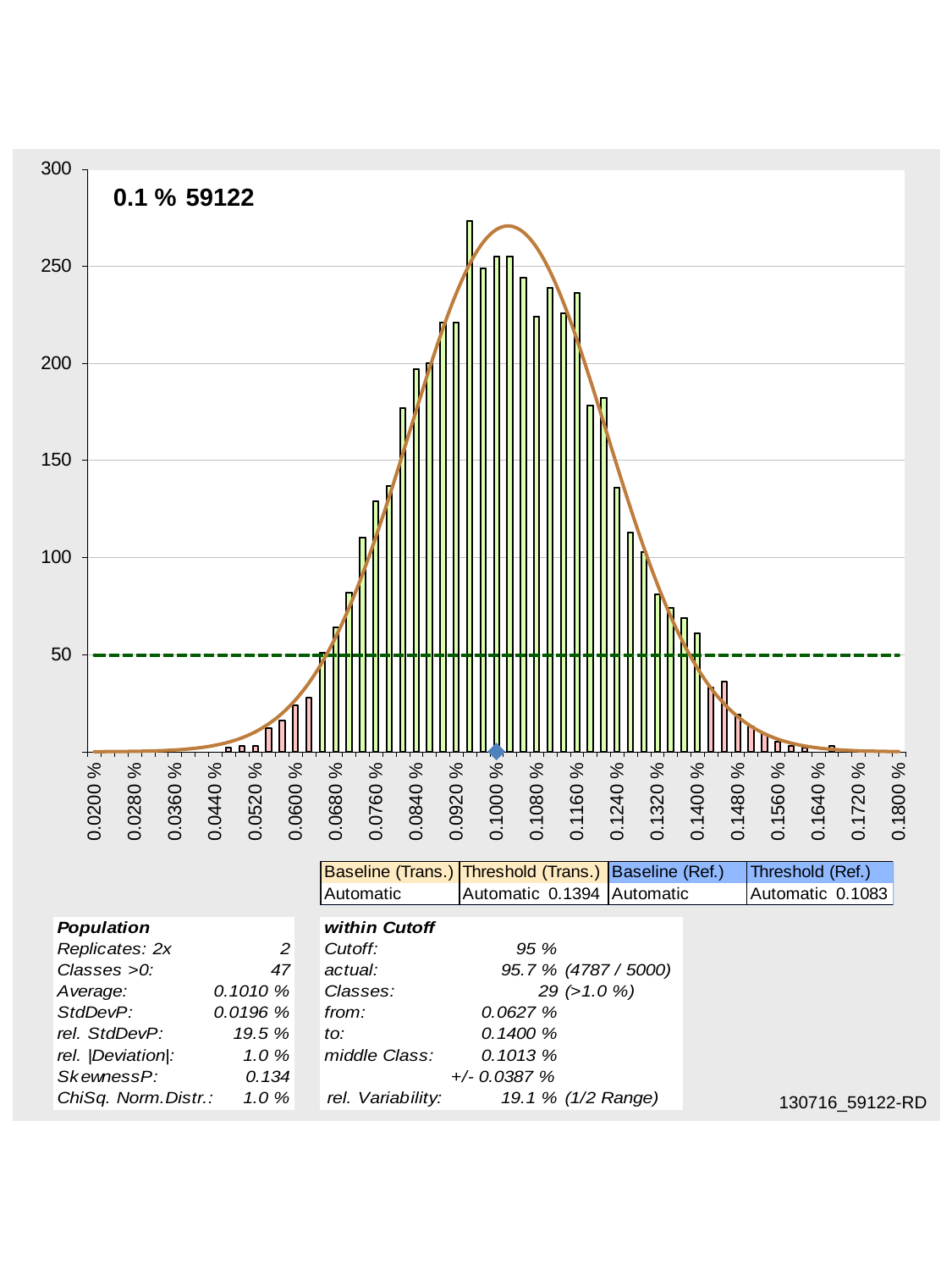

Supplement: Additional file 1: Figure S1. — Frequency distribution for quantification of maize 59122. Exemplary frequency distribution taken from the developed dynamic Excel spreadsheet. Isolated genomic DNA from certified reference material for maize event 59122 (0.1% [w/w]) was quantified with the novel statistical approach. For explanations, see Figure 2. [file 12859_2014_407_MOESM1_ESM.pptx]

## Slide 1
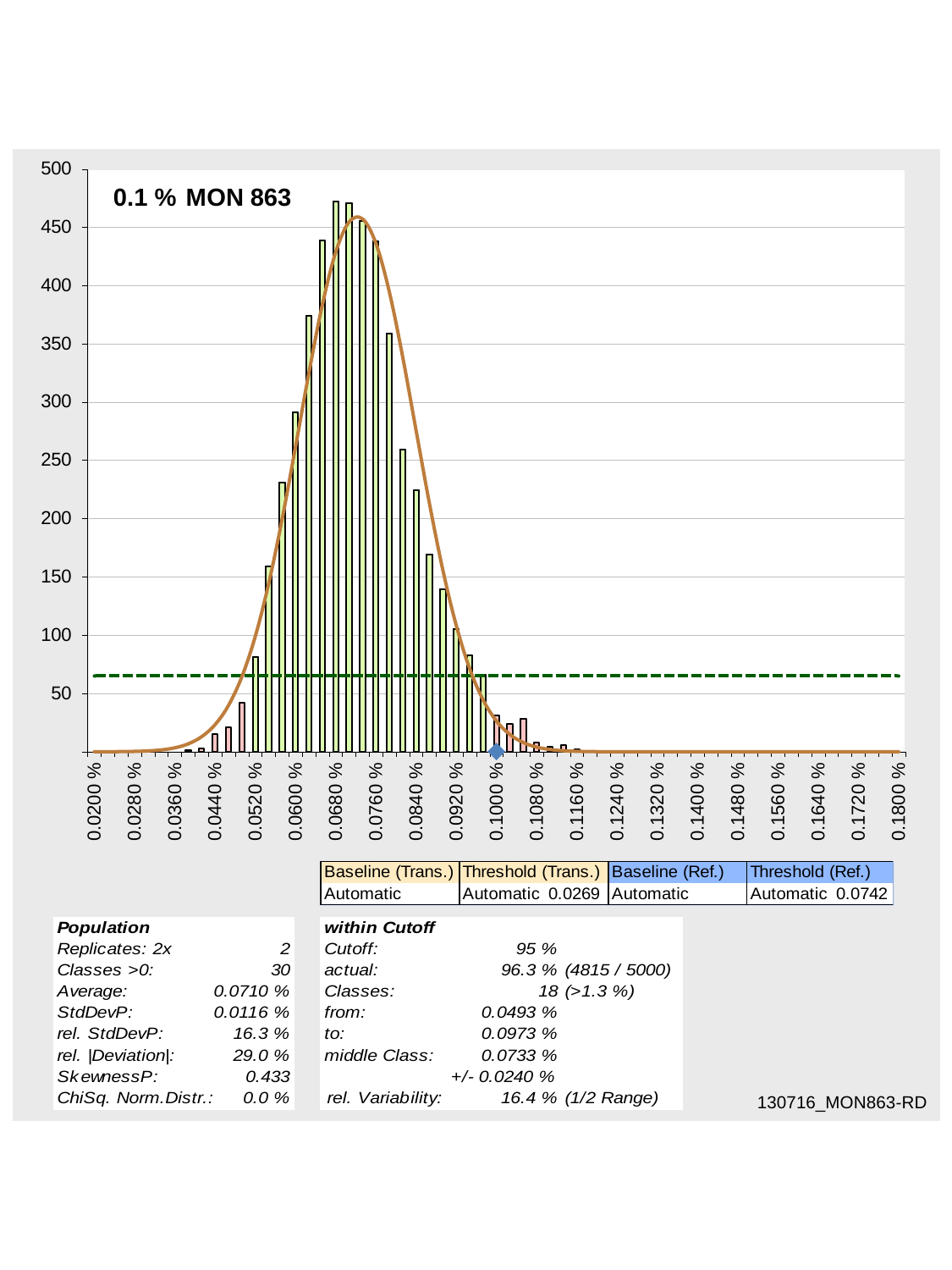

Supplement: Additional file 2: Figure S2. — Frequency distribution for quantification of maize MON 863. Exemplary frequency distribution taken from the developed dynamic Excel spreadsheet. Isolated genomic DNA from certified reference material for maize event MON 863 (0.1% [w/w]) was quantified with the novel statistical approach. For explanations, see Figure 2. [file 12859_2014_407_MOESM2_ESM.pptx]

## Slide 1
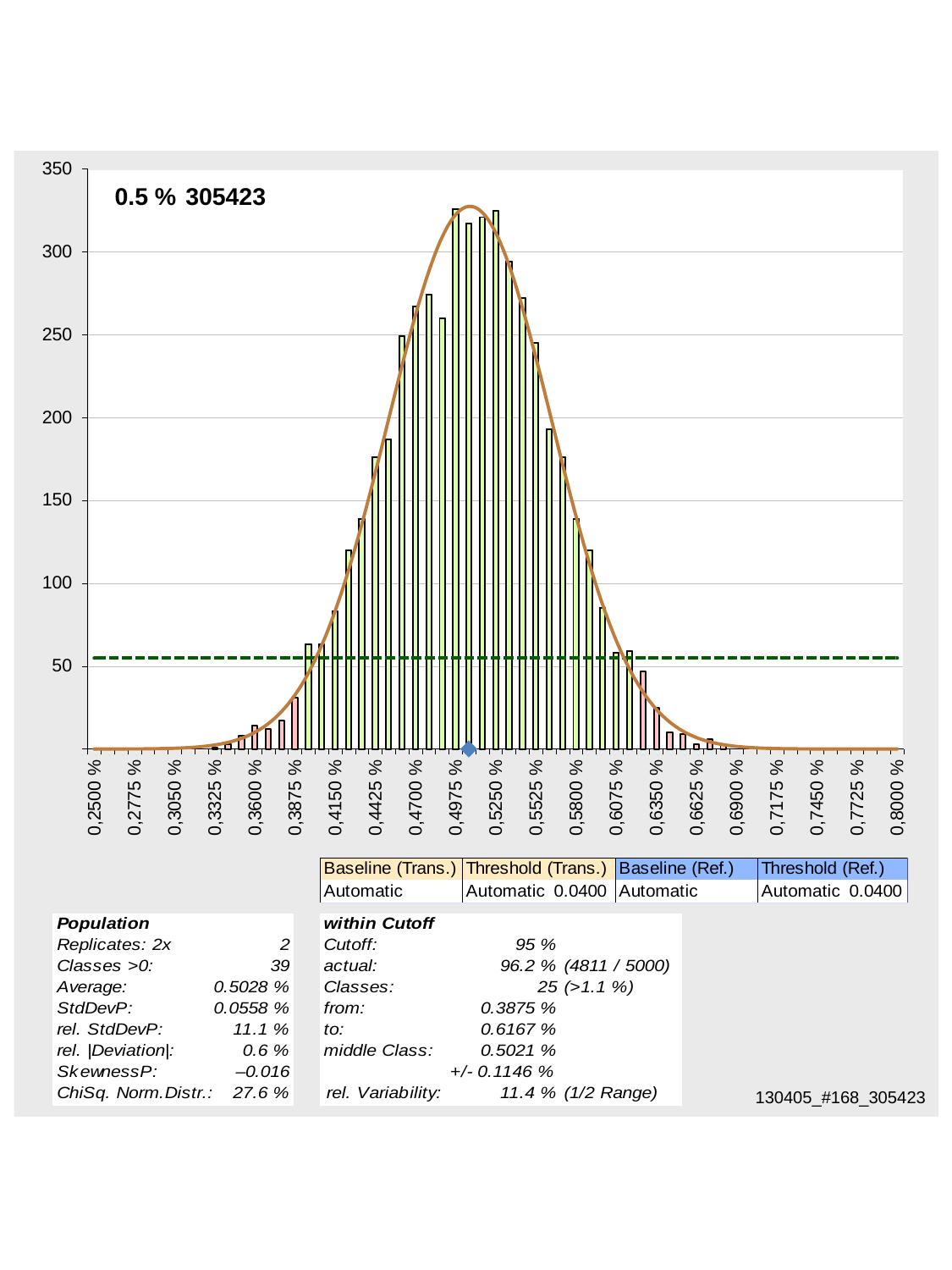

Supplement: Additional file 3: Figure S3. — Frequency distribution for quantification of soy 305423. Exemplary frequency distribution taken from the developed dynamic Excel spreadsheet. Isolated genomic DNA from certified reference material for soy event 305423 (0.5% [w/w]) was quantified with the novel statistical approach. For explanations, see Figure 2. [file 12859_2014_407_MOESM3_ESM.pptx]

## Slide 1
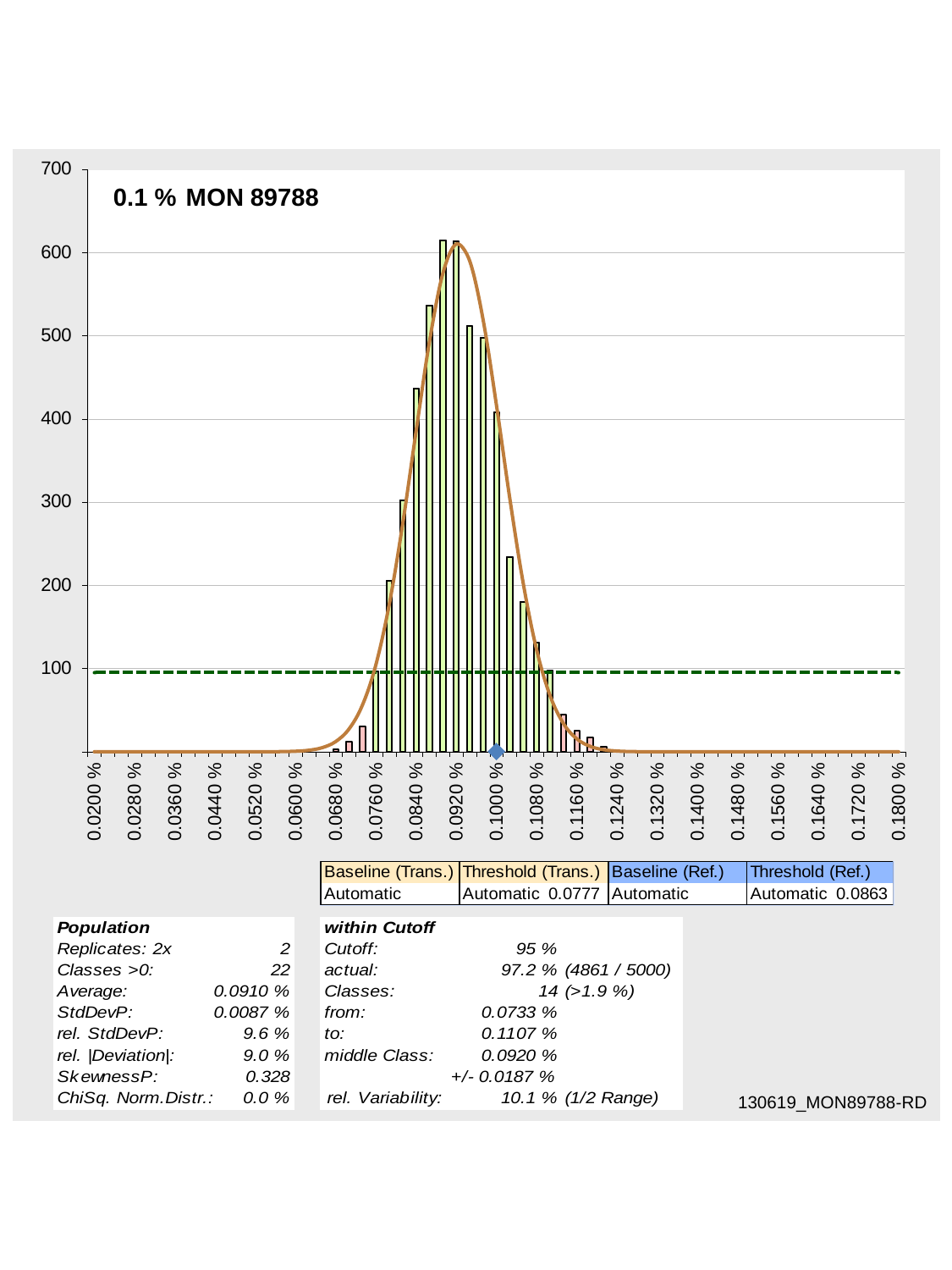

Supplement: Additional file 4: Figure S4. — Frequency distribution for quantification of soy MON 89788. Exemplary frequency distribution taken from the developed dynamic Excel spreadsheet. Isolated genomic DNA from self-mixed reference material for soy event MON 89788 (0.1% [cp/cp]) was quantified with the novel statistical approach. For explanations, see Figure 2. [file 12859_2014_407_MOESM4_ESM.pptx]

## Slide 1
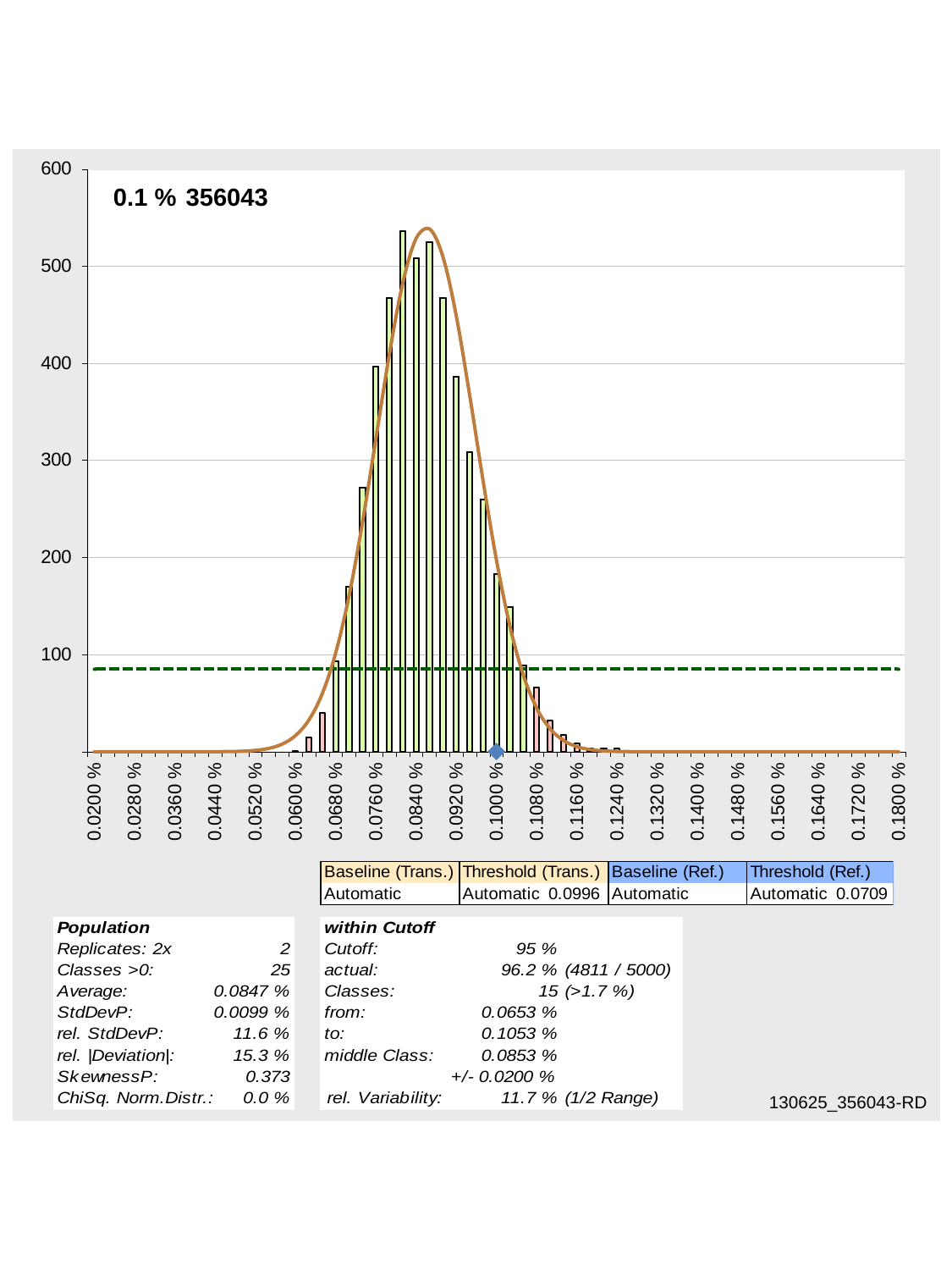

Supplement: Additional file 5: Figure S5. — Frequency distribution for quantification of soy 356043. Exemplary frequency distribution taken from the developed dynamic Excel spreadsheet. Isolated genomic DNA from certified reference material for soy event 356043 (0.1% [w/w]) was quantified with the novel statistical approach. For explanations, see Figure 2. [file 12859_2014_407_MOESM5_ESM.pptx]

## Slide 1
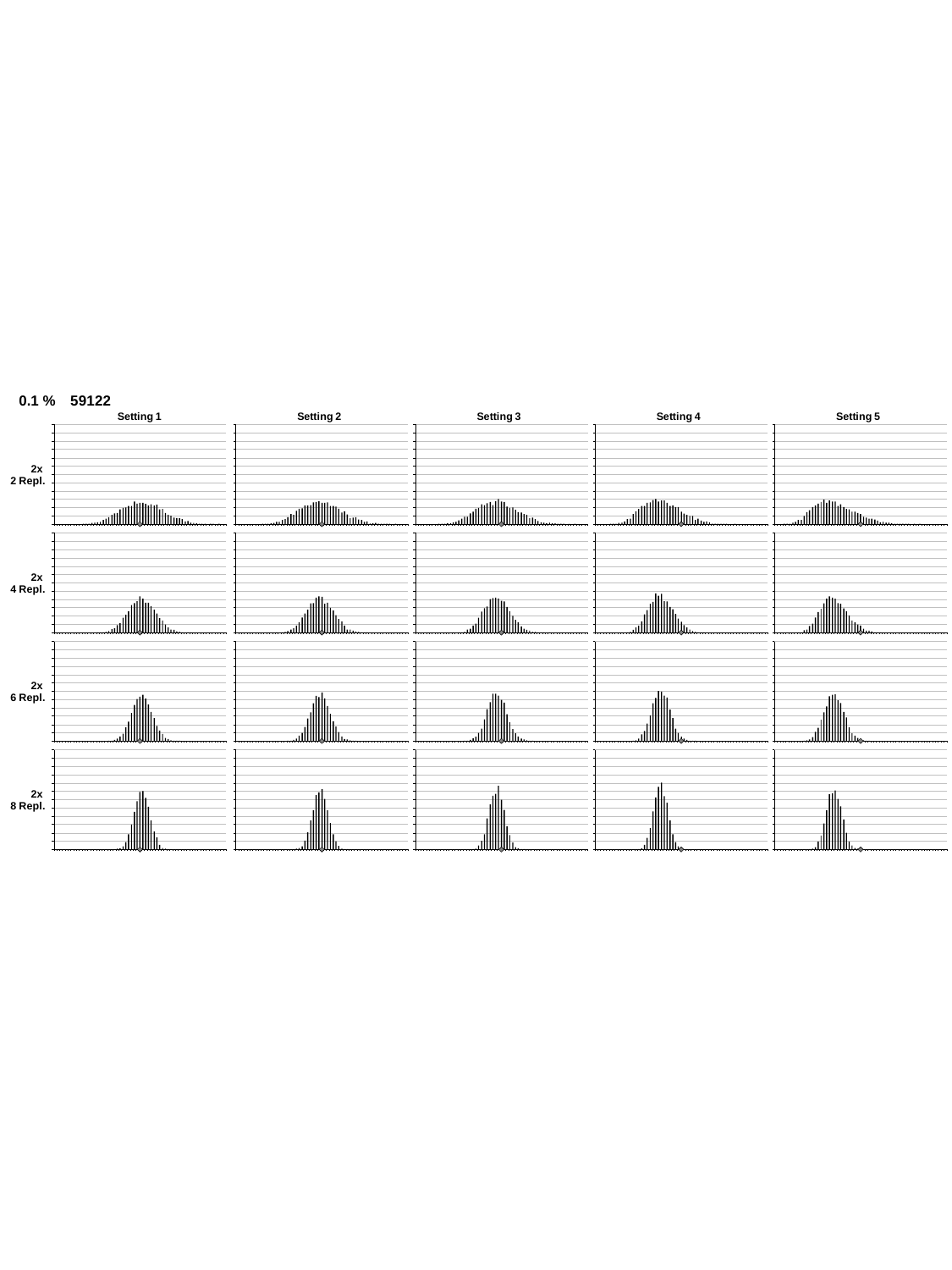

Supplement: Additional file 6: Figure S6. — Frequency distributions for quantification of maize 59122. Comparative overview over 20 exemplary frequency distributions resulting from a single experimental 96-well plate for the quantification of maize event 59122 (compare Additional file 6: Figure S6). Effects of different baseline/threshold settings (Table 1) are shown from left to right, increasing replicate numbers from top to bottom, respectively. The grey diamond marks the nominal GMO content of the reference material. [file 12859_2014_407_MOESM6_ESM.pptx]

## Slide 1
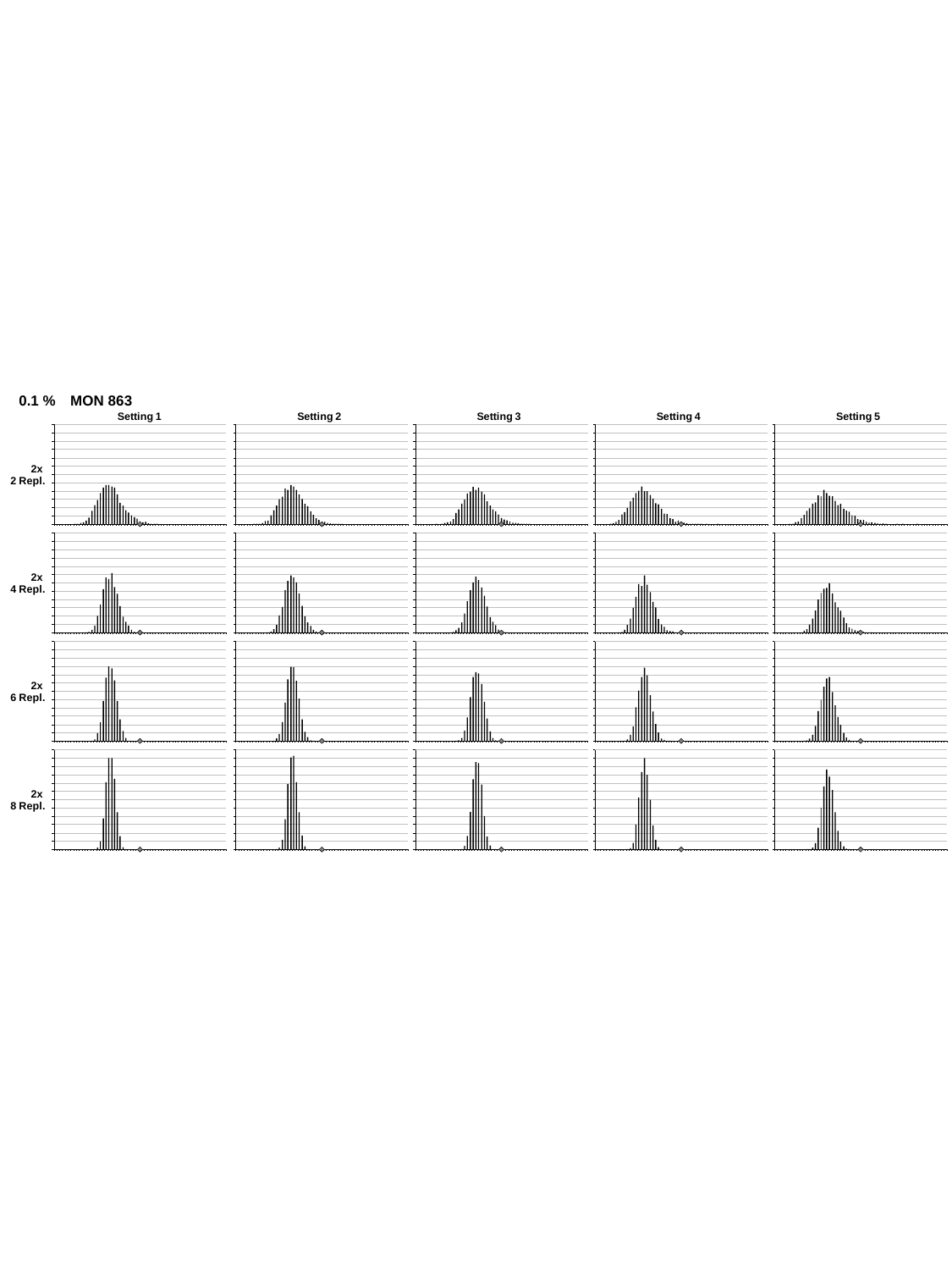

Supplement: Additional file 7: Figure S7. — Frequency distributions for quantification of maize MON 863. Comparative overview over 20 exemplary frequency distributions resulting from a single experimental 96-well plate for the quantification of maize event MON 863 (compare Additional 7: Figure S7). Effects of different baseline/threshold settings (Table 1) are shown from left to right, increasing replicate numbers from top to bottom, respectively. The grey diamond marks the nominal GMO content of the reference material. [file 12859_2014_407_MOESM7_ESM.pptx]

## Slide 1
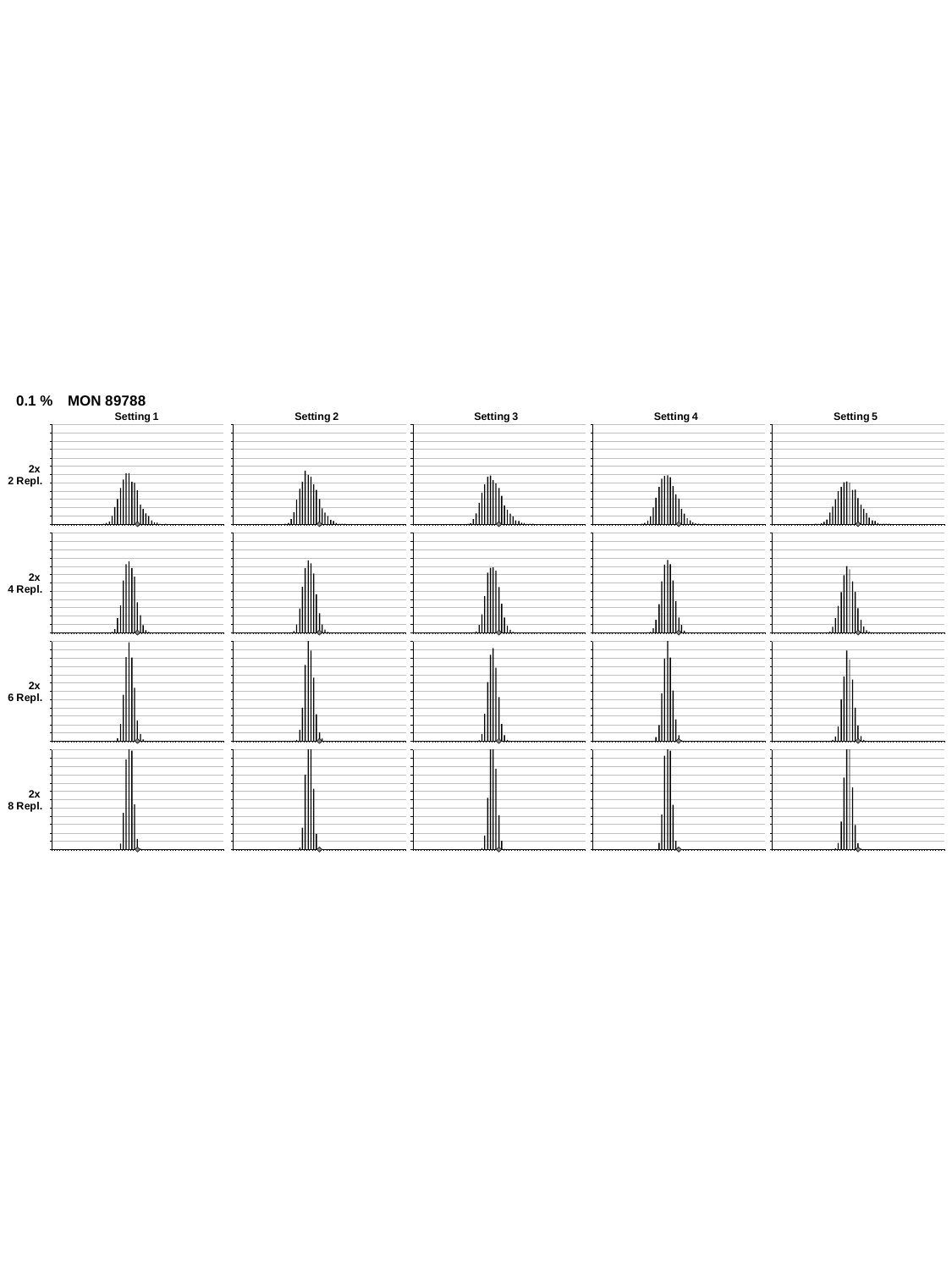

Supplement: Additional file 8: Figure S8. — Frequency distributions for quantification of soy MON 89788. Comparative overview over 20 exemplary frequency distributions resulting from a single experimental 96-well plate for the quantification of soy event MON 89788 (compare Additional file 8: Figure S8). Effects of different baseline/threshold settings (Table 1) are shown from left to right, increasing replicate numbers from top to bottom, respectively. The grey diamond marks the nominal GMO content of the reference material. [file 12859_2014_407_MOESM8_ESM.pptx]

## Slide 1
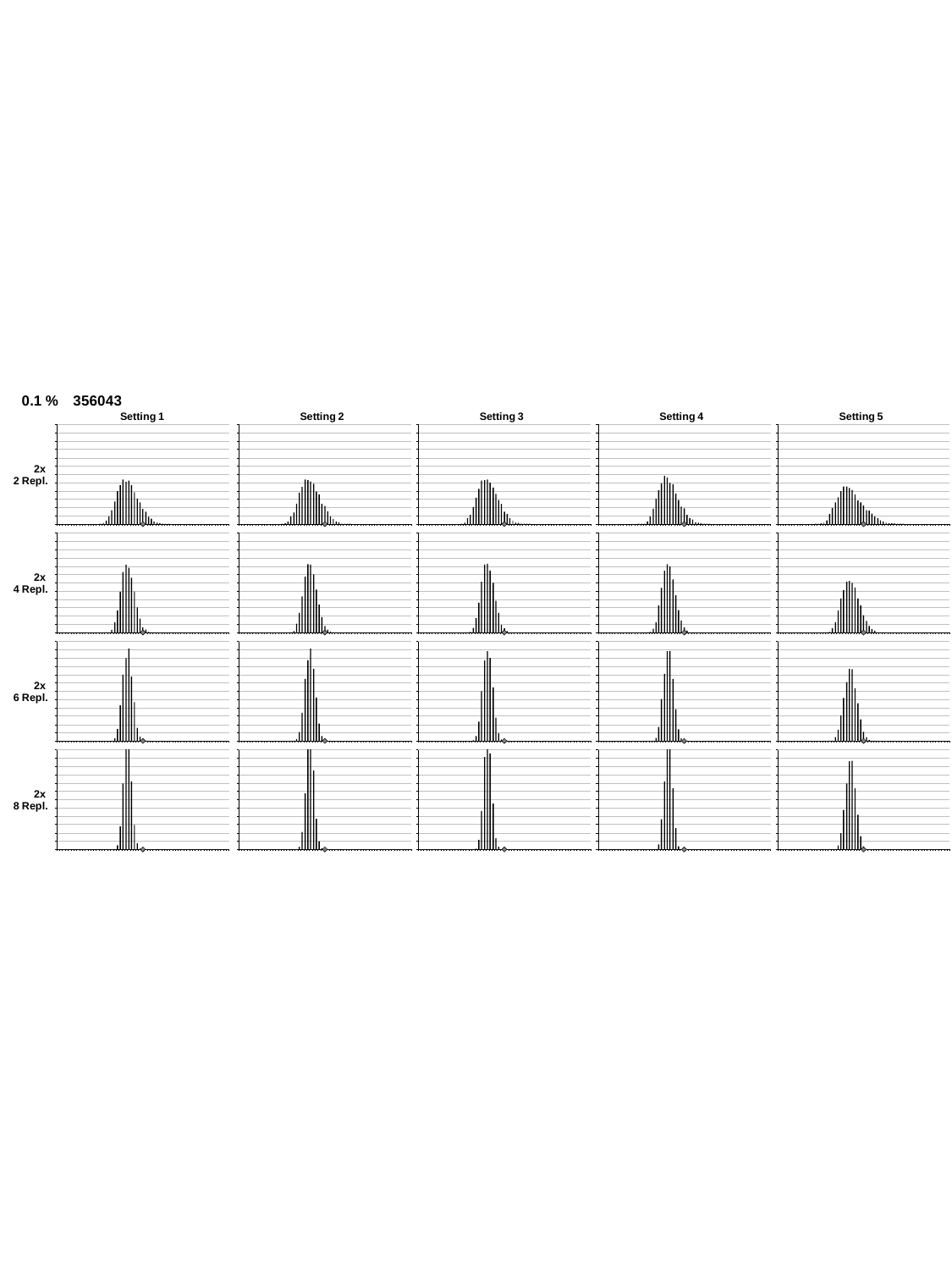

Supplement: Additional file 9: Figure S9. — Frequency distributions for quantification of soy 356043. Comparative overview over 20 exemplary frequency distributions resulting from a single experimental 96-well plate for the quantification of soy event 356043 (compare Additional file 9: Figure S9). Effects of different baseline/threshold settings (Table 1) are shown from left to right, increasing replicate numbers from top to bottom, respectively. The grey diamond marks the nominal GMO content of the reference material. [file 12859_2014_407_MOESM9_ESM.pptx]
