# Supplementary material for: A statistical approach to quantification of genetically modified organisms (GMO) using frequency distributions
Source: BMC Bioinformatics. 2014 Dec 14;15(1):407. doi: 10.1186/s12859-014-0407-x (PMC4279603; doi:10.1186/s12859-014-0407-x)
Supplement: Additional file 10: Table S1. — Empirical skewness (0.5% 305423, Figure 4). Table S2: Empirical skewness (0.1% 59122, Additional file 6: Figure S6). Table S3: Empirical skewness (0.1% MON 863, Additional file 7: Figure S7). Table S4: Empirical skewness (0.1% MON 89788, Additional file 8: Figure S8). Table S5: Empirical skewness (0.1% 356043, Additional file 9: Figure S9). Table S6: $\chi$χ 2 test for normal distribution (0.5% 305423, Figure 4). Table S7: $\chi$χ 2 test for normal distribution (0.1% 59122, Additional file 6: Figure S6). Table S8: $\chi$χ 2 test for normal distribution (0.1% MON 863, Additional file 7: Figure S7). Table S9: $\chi$χ 2 test for normal distribution (0.1% MON 89788, Additional file 8: Figure S8). [file 12859_2014_407_MOESM10_ESM.docx]

Supplementary Table 1 – Empirical skewness (0.5 % 305423, Figure 4)

| Replicates | Setting 1 | Setting 2 | Setting 3 | Setting 4 | Setting 5 |
| --- | --- | --- | --- | --- | --- |
| 2x 2 | -0.016 | -0.021 | -0.040 | 0.026 | 0.083 |
| 2x 4 | -0.010 | -0.033 | -0.024 | 0.006 | 0.056 |
| 2x 6 | 0.037 | 0.024 | 0.032 | 0.027 | 0.054 |
| 2x 8 | 0.034 | 0.032 | 0.035 | 0.029 | 0.031 |

Supplementary Table 2 – Empirical skewness (0.1 % 59122, Supplementary Figure 6)

| Replicates | Setting 1 | Setting 2 | Setting 3 | Setting 4 | Setting 5 |
| --- | --- | --- | --- | --- | --- |
| 2x 2 | 0.134 | 0.153 | 0.214 | 0.330 | 0.510 |
| 2x 4 | 0.104 | 0.113 | 0.148 | 0.203 | 0.303 |
| 2x 6 | 0.069 | 0.070 | 0.109 | 0.065 | 0.119 |
| 2x 8 | 0.014 | 0.016 | 0.058 | 0.050 | 0.070 |

Supplementary Table 3 – Empirical skewness (0.1 % MON 863, Supplementary Figure 7)

| Replicates | Setting 1 | Setting 2 | Setting 3 | Setting 4 | Setting 5 |
| --- | --- | --- | --- | --- | --- |
| 2x 2 | 0.433 | 0.290 | 0.354 | 0.449 | 0.552 |
| 2x 4 | 0.210 | 0.145 | 0.180 | 0.265 | 0.349 |
| 2x 6 | 0.098 | 0.062 | 0.088 | 0.131 | 0.185 |
| 2x 8 | 0.037 | 0.045 | 0.055 | 0.102 | 0.138 |

Supplementary Table 4 – Empirical skewness (0.1 % MON 89788, Supplementary Figure 8)

| Replicates | Setting 1 | Setting 2 | Setting 3 | Setting 4 | Setting 5 |
| --- | --- | --- | --- | --- | --- |
| 2x 2 | 0.328 | 0.392 | 0.467 | 0.261 | 0.242 |
| 2x 4 | 0.113 | 0.137 | 0.165 | 0.083 | 0.076 |
| 2x 6 | 0.114 | 0.127 | 0.140 | 0.087 | 0.077 |
| 2x 8 | 0.037 | 0.040 | 0.048 | 0.032 | 0.029 |

Supplementary Table 5 – Empirical skewness (0.1 % 356043, Supplementary Figure 9)

| Replicates | Setting 1 | Setting 2 | Setting 3 | Setting 4 | Setting 5 |
| --- | --- | --- | --- | --- | --- |
| 2x 2 | 0.373 | 0.340 | 0.324 | 0.420 | 0.532 |
| 2x 4 | 0.237 | 0.211 | 0.222 | 0.186 | 0.268 |
| 2x 6 | 0.089 | 0.080 | 0.096 | 0.056 | 0.112 |
| 2x 8 | 0.033 | 0.033 | 0.044 | -0.013 | 0.017 |

Supplementary Table 6 – χ^2^ test for normal distribution (0.5 % 305423, Figure 4)

| Replicates | Setting 1 | Setting 2 | Setting 3 | Setting 4 | Setting 5 |
| --- | --- | --- | --- | --- | --- |
| 2x 2 | 27.6 % | 47.3 % | 28.2 % | 52.7 % | 51.0 % |
| 2x 4 | 58.0 % | 83.4 % | 28.3 % | 89.6 % | 11.0 % |
| 2x 6 | 98.2 % | 89.0 % | 76.3 % | 95.8 % | 85.2 % |
| 2x 8 | 57.6 % | 19.0 % | 97.4 % | 99.2 % | 77.2 % |

Supplementary Table 7 – χ^2^ test for normal distribution (0.1 % 59122, Supplementary Figure 6)

| Replicates | Setting 1 | Setting 2 | Setting 3 | Setting 4 | Setting 5 |
| --- | --- | --- | --- | --- | --- |
| 2x 2 | 1.0 % | 0.0 % | 0.0 % | 0.0 % | 0.0 % |
| 2x 4 | 20.4 % | 2.8 % | 2.8 % | 0.0 % | 0.0 % |
| 2x 6 | 94.7 % | 75.0 % | 30.5 % | 60.9 % | 7.4 % |
| 2x 8 | 91.6 % | 58.2 % | 1.1 % | 39.7 % | 25.2 % |

Supplementary Table 8 – χ^2^ test for normal distribution (0.1 % MON 863, Supplementary Figure 7)

| Replicates | Setting 1 | Setting 2 | Setting 3 | Setting 4 | Setting 5 |
| --- | --- | --- | --- | --- | --- |
| 2x 2 | 0.0 % | 0.0 % | 0.0 % | 0.0 % | 0.0 % |
| 2x 4 | 0.0 % | 1.8 % | 0.0 % | 0.0 % | 0.0 % |
| 2x 6 | 2.3 % | 55.9 % | 7.8 % | 0.0 % | 0.3 % |
| 2x 8 | 0.5 % | 12.1 % | 2.3 % | 2.4 % | 1.6 % |

Supplementary Table 9 – χ^2^ test for normal distribution (0.1 % MON 89788, Supplementary Figure 8)

| Replicates | Setting 1 | Setting 2 | Setting 3 | Setting 4 | Setting 5 |
| --- | --- | --- | --- | --- | --- |
| 2x 2 | 0.0 % | 0.0 % | 0.0 % | 0.0 % | 0.0 % |
| 2x 4 | 0.0 % | 0.0 % | 0.0 % | 1.7 % | 11.3 % |
| 2x 6 | 0.7 % | 0.1 % | 0.3 % | 5.8 % | 3.3 % |
| 2x 8 | 0.2 % | 0.0 % | 0.1 % | 0.7 % | 37.9 % |

Supplementary Table 10 – χ^2^ test for normal distribution (0.1 % 356043, Supplementary Figure 9)

| Replicates | Setting 1 | Setting 2 | Setting 3 | Setting 4 | Setting 5 |
| --- | --- | --- | --- | --- | --- |
| 2x 2 | 0.0 % | 0.0 % | 0.0 % | 0.0 % | 0.0 % |
| 2x 4 | 0.0 % | 0.0 % | 0.0 % | 0.0 % | 0.0 % |
| 2x 6 | 0.1 % | 1.2 % | 1.3 % | 0.8 % | 3.3 % |
| 2x 8 | 19.6 % | 9.4 % | 0.5 % | 0.1 % | 0.4 % |
